# Hematopoietic Stem Cells as a Novel Source of Dental Tissue Cells

**DOI:** 10.1038/s41598-018-26258-y

**Published:** 2018-05-23

**Authors:** Katie R. Wilson, In-Hong Kang, Uday Baliga, Ying Xiong, Shilpak Chatterjee, Emily Moore, Beneta Parthiban, Krishnamurthy Thyagarajan, James L. Borke, Shikhar Mehrotra, Keith L. Kirkwood, Amanda C. LaRue, Makio Ogawa, Meenal Mehrotra

**Affiliations:** 1 Department of Pathology and Laboratory Medicine, Medical University of South Carolina, Charleston, SC 29425 USA; 2 Department of Surgery, Medical University of South Carolina, Charleston, SC 29425 USA; 3 Department of Oral Health Sciences, Medical University of South Carolina, Charleston, SC 29425 USA; 4 College of Dental Medicine, Western University of Health Sciences, Pomona, CA 91766 USA; 5 Department of Oral Biology, University at Buffalo, The State University of New York, Department of Oral Oncology, Roswell Park Comprehensive Cancer Center, Buffalo, NY 14260 USA; 6 Hollings Cancer Center, Medical University of South Carolina, Charleston, SC 29425 USA; 7 Ralph H Johnson VA Medical Center, Charleston, SC 29425 USA; 8 Center for Oral Health Research, Medical University of South Carolina, Charleston, SC 29425 USA

## Abstract

While earlier studies have suggested that cells positive for hematopoietic markers can be found in dental tissues, it has yet to be confirmed. To conclusively demonstrate this, we utilized a unique transgenic model in which all hematopoietic cells are green fluorescent protein^+^ (GFP^+^). Pulp, periodontal ligament (PDL) and alveolar bone (AvB) cell culture analysis demonstrated numerous GFP^+^ cells, which were also CD45^+^ (indicating hematopoietic origin) and co-expressed markers of cellular populations in pulp (dentin matrix protein-1, dentin sialophosphoprotein, alpha smooth muscle actin [ASMA], osteocalcin), in PDL (periostin, ASMA, vimentin, osteocalcin) and in AvB (Runx-2, bone sialoprotein, alkaline phosphatase, osteocalcin). Transplantation of clonal population derived from a single GFP^+^ hematopoietic stem cell (HSC), into lethally irradiated recipient mice, demonstrated numerous GFP^+^ cells within dental tissues of recipient mice, which also stained for markers of cell populations in pulp, PDL and AvB (used above), indicating that transplanted HSCs can differentiate into cells in dental tissues. These hematopoietic-derived cells deposited collagen and can differentiate in osteogenic media, indicating that they are functional. Thus, our studies demonstrate, for the first time, that cells in pulp, PDL and AvB can have a hematopoietic origin, thereby opening new avenues of therapy for dental diseases and injuries.

## Introduction

Loss of teeth resulting from decay, periodontal diseases, trauma, or surgery negatively affects quality of life. During recent years, the quest for identifying the ideal stem cell to regenerate tooth has attracted increased attention. Earlier studies have shown that cells in bone marrow, which contains both hematopoietic stem cells (HSCs) and mesenchymal stem cells (MSCs), can differentiate into odontoblast-like cells^1,2^ and regenerate dental pulp^3^. Recently, it has been shown that compressive forces in the scaffolds can induce adult bone marrow stem cells to undergo a lineage switch and begin to form dentin-like tissue^4^. Local transplantation of bone marrow cells regenerated periodontal ligament (PDL)^5–8^, and their migration after systemic transplantation into periodontal tissues was increased by mechanical stress^9^. Enhanced green fluorescent protein (EGFP)-expressing cells were observed around periodontal defects after systemic transplantation of bone marrow derived cells^10,11^, which were capable of participating in tissue repair^12^. GFP^+^ bone marrow cells have been shown to differentiate into dental-specific cells and expressed dental-specific proteins after systemic transplantation^13^.

Bone marrow also includes the HSCs which till now are said to only give rise to blood cells and some tissue cells such as osteoclasts. However, recent studies (stated below) have begun to suggest the plasticity of HSCs (ability to give rise to other cells). Using a transplantation technique in which bone marrow of lethally irradiated mice is replaced with a clonal population derived from a single GFP^+^ HSC, we have previously shown that a number of fibroblasts/myofibroblasts in multiple tissues^14–16^, adipocytes^17^ and osteo-chondrocytes^18,19^ are derived from HSCs. In fact, in previous studies in the dental tissue, CD34^+^ (marker for HSCs) cells have been demonstrated in the healthy human gingiva^20^ and majority of GFP^+^ cells were CD45^+^ (pan hematopoietic marker) in reparative dentin in a parabiosis model^21^, suggesting that HSC-derived cells may also be present in the dental tissues.

In this study, we demonstrate, for the first time, that cells having a hematopoietic origin are present in the dental tissues. We also establish that after systemic transplantation of lethally irradiated mice with a clonal population derived from a single HSC, HSC-derived cells expressing markers of resident cellular populations can be detected in the pulp, PDL and alveolar bone (AvB) of the recipient mice. We also show that these cells can deposit collagen and undergo osteogenic differentiation, depositing calcium *in vitro*. Together with the above reports, our studies clearly delineate that hematopoietic-derived cells are present in the pulp, PDL and AvB, thereby providing a novel source of cells in the dental region.

## Results

### CD45^+^ cells are observed in pulp, PDL and AvB

**Figure 1:**
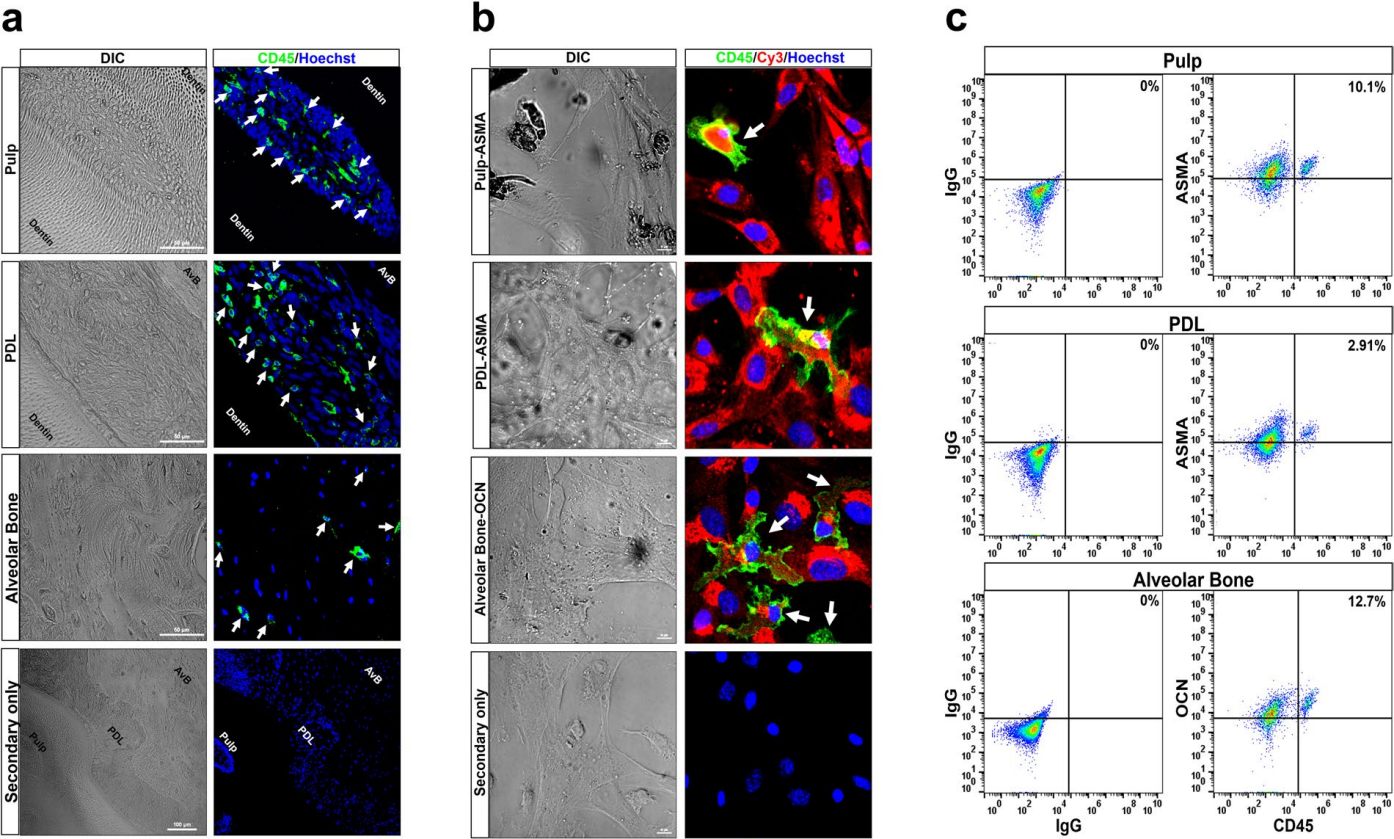
CD45^+^ cells are observed in pulp, PDL and AvB: (**a**) Sections through the molar crown from a C57BL/6 mouse were taken and immunofluorescent staining was conducted using CD45 antibody and Alexa Fluor 488 secondary antibody. CD45 positive cells (green staining, white arrows) can be observed in all the three tissues, pulp, PDL and AvB. CD45 positive staining can be seen mainly in the pulp cells, CD45^+^ cells are present all along the length of the PDL and CD45^+^ osteocytes can be seen in the bone matrix. Thus, cells with a hematopoietic origin are present in the dental tissues. Bar: pulp, PDL and AvB = 50 µM; secondary only = 100 µM. (**b**) To determine if the cells with the hematopoietic origin are fibroblasts and osteoblasts (major population of cells found in these tissues), cells were extracted from the pulp, PDL and AvB and cultured for 7–14 days (n = 3). They were then stained with CD45 and ASMA antibodies (for pulp and PDL) and CD45 and osteocalcin (OCN) antibodies (for AvB) and Alexa Fluor 488 and Cy3 secondary antibodies. Immunofluorescent imaging demonstrates the presence of CD45 staining (green) in the membranes of some of the cells which stained positive for ASMA (red) in pulp and PDL and OCN (red) in AvB (white arrows), indicating the existence of hematopoietic-derived fibroblasts and osteoblasts respectively. Bar for all = 10 µM. (**c**) These cells were further analyzed by flow cytometry, for the expression of CD45 and ASMA (in pulp and PDL) and CD45 and OCN (in AvB), to determine the percentage of fibroblasts and osteoblasts which have a hematopoietic origin. In this representative image, about 10% of pulp cells and 3% of PDL cells were found to express both CD45 and ASMA. In AvB, about 13% of cells were positive for CD45 and OCN. This indicates that there is a percentage of fibroblasts in pulp and PDL and a percentage of osteoblasts/osteocytes in AvB which have a hematopoietic origin.

To test the hypothesis that cells with a hematopoietic origin could be present in pulp, PDL and AvB, we took sections through the molar crown of C57/BL6 mice and conducted immunofluorescent staining using CD45 antibody. A number of CD45^+^ cells (indicated by green staining and marked by arrows), having the morphology of fibroblasts and osteocytes, can be visualized within the pulp, PDL and AvB (Fig. 1a). CD45 positive staining can be seen mainly in the pulp cells, CD45^+^ cells are present all along the length of the PDL and CD45^+^ osteocytes can be seen in the bone matrix. To further confirm that these CD45^+^ cells were indeed fibroblasts and osteoblasts, the major resident cell populations of these regions and not immune cells, cells from all the three tissues were cultured for 7–14 days before being stained for CD45 and alpha smooth muscle actin (ASMA) for pulp and PDL and CD45 and osteocalcin for AvB. The cells were then either visualized by immunofluorescent staining or analyzed by flow cytometry (n = 3). Figure 1b shows the immunofluorescent staining of CD45 (in green) and either ASMA or osteocalcin (in red) in the cultured cells from pulp, PDL and AvB. CD45 staining can be observed (marked by arrows) in the membranes of some of the cells which also stained positive for ASMA in pulp and PDL and for osteocalcin in AvB, indicating the presence of hematopoietic-derived fibroblasts and osteoblasts respectively. On analyzing by flow cytometry, about 5–18% of pulp and 3–5% of PDL cells were found to be CD45^+^ and ASMA^+^ while in AvB, about 12–20% of cells were CD45^+^ and osteocalcin^+^, again indicating the percentage of fibroblast and osteoblasts which have a hematopoietic origin (Fig. 1c). Thus, these data strongly suggest that cells of hematopoietic origin are present in pulp, PDL and AvB of mice.

### Presence of hematopoietic derived cells in pulp, PDL and AvB in transgenic mice

**Figure 2:**
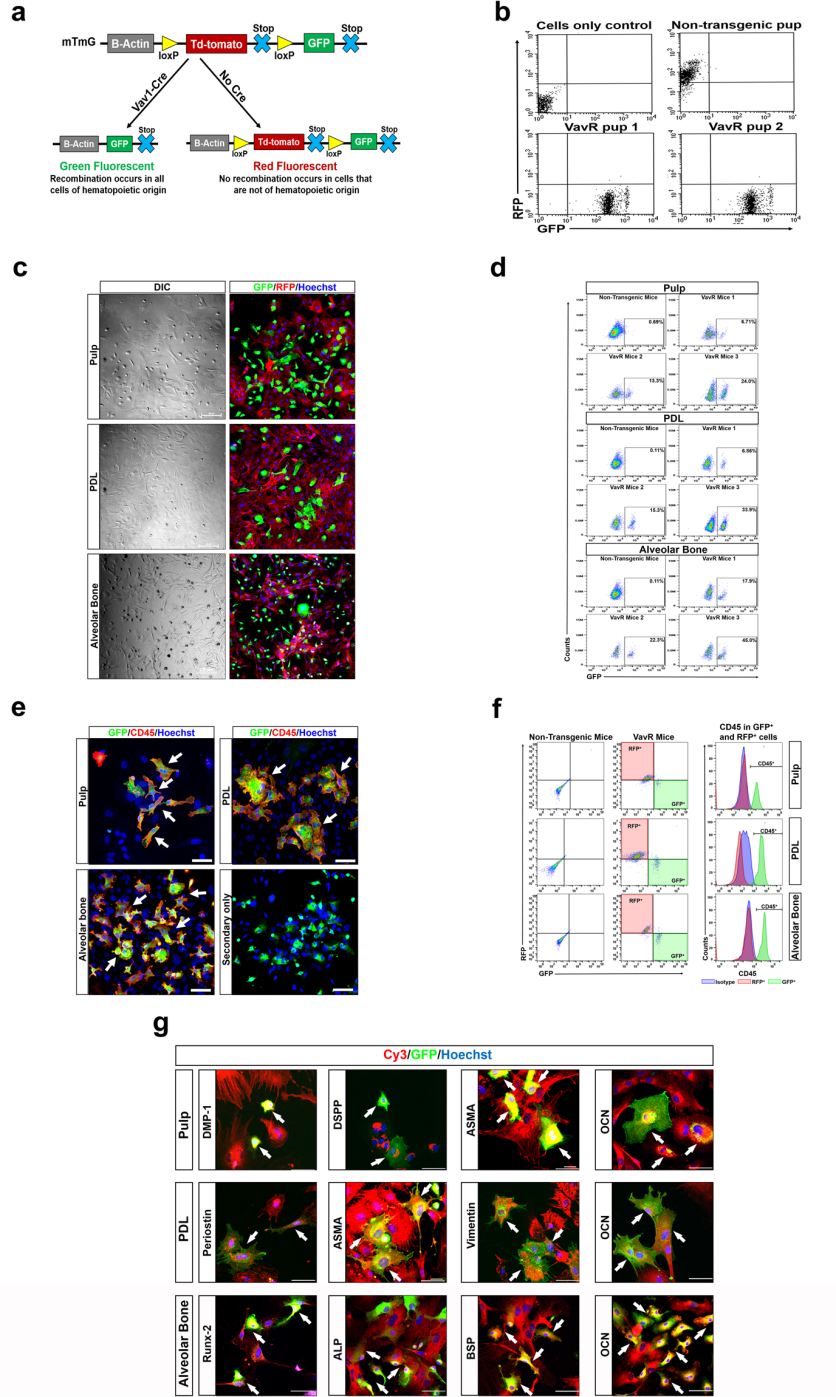
Presence of hematopoietic-derived cells in pulp, PDL and AvB in transgenic mice: (**a**) Generation of Cre-activated dual fluorescence mouse (VavR), in which all hematopoietic cells are GFP^+^ (after Cre-mediated excision of floxed RFP) while all non-hematopoietic cells, with no Cre activity, are RFP^+^. (**b**) Flow cytometric analysis of peripheral blood from 4 weeks old VavR pups show presence of GFP^+^ cells while non-transgenic pup demonstrates presence of RFP^+^ cells. (**c**) Cultured cells from pulp, PDL and AvB, from VavR mice, were analyzed by immunofluorescence. GFP^+^ cells (hematopoietic-derived) can be appreciated among RFP^+^ cells (stromal-derived). Bar = 100 µm. (**d**) Representative flow cytometric analysis from 3 mice show that percentage of GFP^+^ cells ranged from 6–24% in pulp, 6–34% in PDL and 17–50% in AvB, indicating their hematopoietic origin. (**e**) Immunofluorescent staining for CD45 (red; Cy5) was performed to confirm hematopoietic origin of GFP^+^ cells. Only GFP^+^ cells, from all three tissues, stained positive for CD45 (red staining on cell surface). Bar = 50 µm. (**f**) Flow cytometric analysis of pulp, PDL and AvB cells after staining with CD45-APC. Analyses was performed after initially gating for GFP^+^ (green) and RFP^+^ (red) cells and examining expression of CD45 in these gated populations. Representative images confirm that all GFP^+^ cells from pulp, PDL and AvB are also CD45^+^ (green graph) while all RFP^+^ cells are CD45^−^ (red graph). Isotype for CD45 (negative control) is shown by blue graph. (**g**) To examine the resident cell population in the different tissues which was hematopoietic-derived, immunofluorescent staining was conducted for various markers of these cells using DMP-1, DSPP, ASMA and osteocalcin for pulp, periostin, ASMA, vimentin and osteocalcin for PDL and Runx-2, BSP, ALP and osteocalcin for AvB (red; Cy5). All GFP^+^ cells (green) also stained for the various markers (yellow cells; white arrows) in pulp, PDL and AvB, indicating that GFP^+^ cells also expressed the markers of the resident populations in these tissues. Bar = 50 µm. In all images, Hoechst shows the nuclear staining (blue) while DIC images shows morphology.

In order to conclusively demonstrate the hematopoietic origin of the cells in the pulp, PDL and AvB, we utilized a specific transgenic mice first described by Suga *et al*.^22^. They reported the generation of a Cre-activated dual fluorescence mouse (VavR) with the Cre expression being under the Vav-1 gene promoter. Vav-1 is a pan-hematopoietic marker expressed exclusively in hematopoietic system^23^. In VavR mice, obtained by crossbreeding Vav-Cre and mTmG strains, all hematopoietic cells, which express Vav-1, are labeled with GFP (after a Cre-mediated excision of floxed RFP) while all non-hematopoietic cells, with no Cre activity, remain RFP labeled (Fig. 2a). Importantly, if Vav-1 has been expressed in a cell at any point during development, that cell is permanently labeled with GFP which eliminates the problem of transient expression of hematopoietic lineage markers such as CD45^22^. In order to differentiate transgenic VavR pups from the non-transgenic pups, we analyzed their peripheral blood by flow cytometry. Representative images from the analysis shows the presence of GFP^+^ cells in the peripheral blood of transgenic VavR pups while the non-transgenic pup demonstrates RFP^+^ cells only (Fig. 2b). After confirming transgenic pups, we cultured the cells from pulp, PDL and AvB of these VavR mice and analyzed them by immunofluorescence and flow cytometry. GFP^+^ cells can be detected among the RFP^+^ cells in cultures from all three dental tissues (Fig. 2c). Flow cytometric analysis (n = 7 mice) demonstrated that the percentage of the GFP^+^ cells ranged from 6–24% in pulp, from 6–34% in PDL and from 17–50% in AvB (Fig. 2d). The above data indicate the presence of hematopoietic-derived cells among the stromal-derived cells in the pulp, PDL and AvB. To confirm that only the GFP^+^ cells were of hematopoietic origin, we stained the cells from pulp, PDL and AvB with CD45. Figure 2e demonstrates that only GFP^+^ cells, from all three tissues, stained positive for CD45 (seen as red staining on the cell surface). This is confirmed by flow cytometric analysis showing that all GFP^+^ cells from pulp, PDL and AvB stained positive for CD45 also while RFP^+^ cells do not show any CD45 staining (Fig. 2f). Lastly we wanted to confirm which resident cells populations in the pulp, PDL and AvB had a hematopoietic origin. The main cell populations in pulp are fibroblasts and odontoblasts while PDL consists of fibroblasts, bone and cementum cells and osteoblasts/osteocytes form the main cellular population in AvB. Thus, we used markers such as dentin matrix protein-1 (DMP-1), dentin sialophosphoprotein (DSPP), ASMA, vimentin, periostin, runt-related transcription factor 2 (Runx-2), bone sialoprotein (BSP), alkaline phosphatase (ALP) and osteocalcin to identify the cell populations as has been described in studies previously^24–30^. As seen by immunofluorescent staining, almost all of the GFP^+^ cells in pulp also stained for DMP-1, DSPP, ASMA and osteocalcin, in PDL also stained for periostin, ASMA, vimentin and osteocalcin and in AvB also stained positive for Runx-2, BSP, ALP and osteocalcin (as indicated by dual stained yellow cells) (Fig. 2g). Thus, these data conclusively establish that cells with a hematopoietic origin are present within the resident cell populations of pulp, PDL and AvB.

### CD45 enriched bone marrow cells give rise to cells in the pulp, PDL and AvB

**Figure 3:**
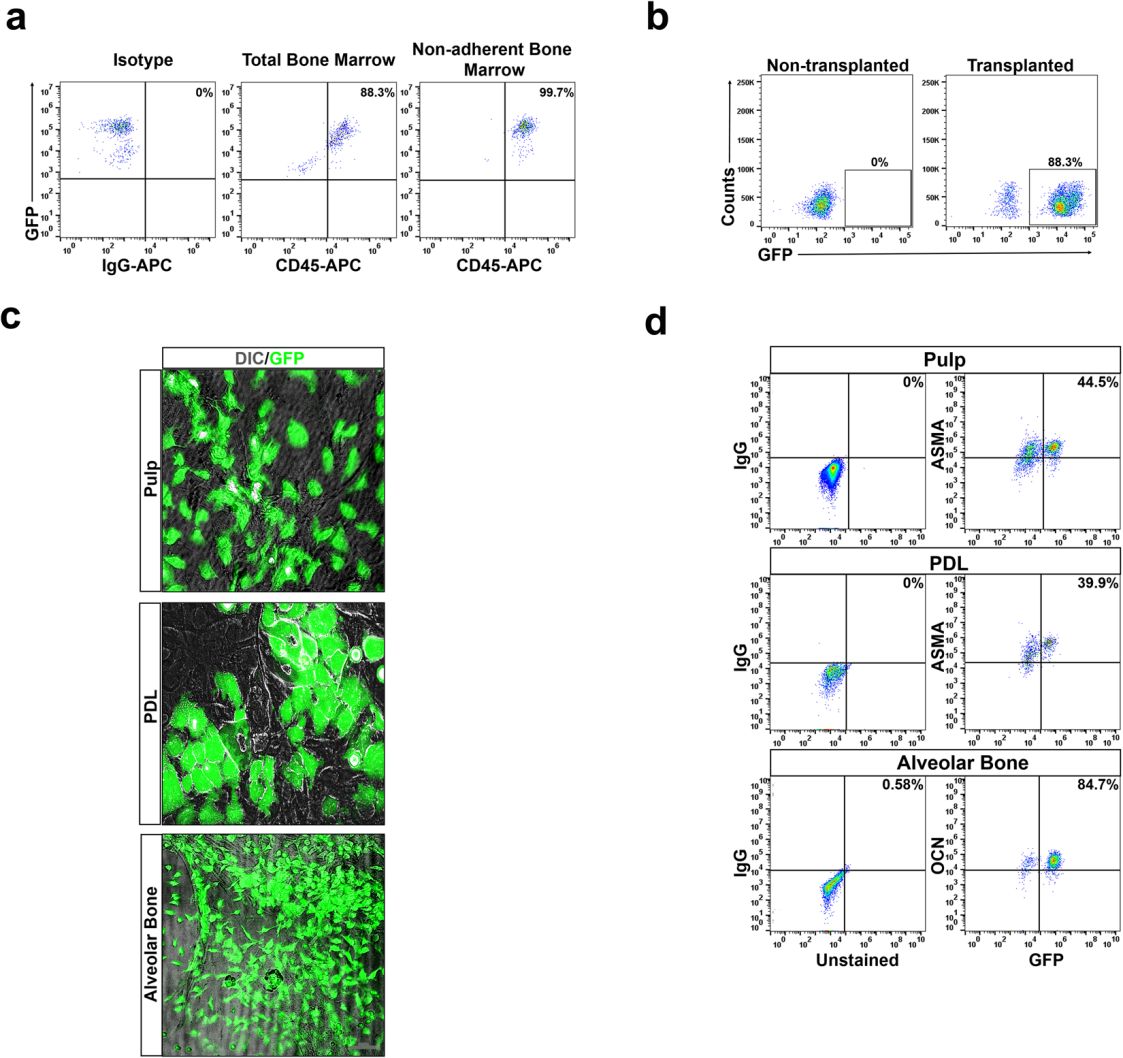
CD45 enriched bone marrow cells give rise to cells in the pulp, PDL and AvB: (**a**) Bone marrow cells from GFP mice were cultured for 4 days and supernatant with non-adherent cells (NACs) was collected. NACs were analyzed by flow cytometry for CD45^+^ cells. Representative images show that almost 100% of the NACs, after 4 days in culture, were CD45^+^ indicating complete enrichment of the population. (**b**) To check for engraftment in the recipient mice, peripheral blood from lethally irradiated non-GFP recipient mice, transplanted with the CD45 enriched NACs from GFP mice, was analyzed by flow cytometry, one month after transplantation, for the presence of GFP^+^ cells. Representative image shows a mouse with an engraftment of 88%. (**c**) Pulp, PDL and AvB cells were extracted from the engrafted mice and cells cultured for 2 weeks, fixed and observed under microscope for presence of GFP^+^ cells. Superimposition of GFP images with DIC images (showing morphology), demonstrates numerous GFP^+^ cells in the cultures from all three dental tissues present among GFP^−^ resident cells. Bar = 100 µm. (**d**) The cells from all three tissues (n = 3) were analyzed by flow cytometry, for the percentage of GFP^+^ cells. Representative images show that the percentage of GFP^+^ cells were 44.5% in pulp, 39.9% in PDL and 84.7% in AvB. Cells from pulp. PDL and AvB, from a non-transplanted non-GFP mouse were taken as controls.

Next we wanted to confirm if the HSCs in the bone marrow have the ability to give rise to the cells in the pulp, PDL and AvB. In order to initiate these studies, we first transplanted mice with plastic non-adherent bone marrow cells. Studies have shown that HSCs reside in the CD45 enriched non-adherent cell (NAC) population of bone marrow^31^. We performed initial experiments by transplanting GFP^+^ NACs into lethally irradiated mice. Almost 100% of bone marrow NACs were CD45^+^ after 4 days in culture indicating complete enrichment of this population (Fig. 3a). This population was then collected and transplanted into lethally irradiated mice. Peripheral blood analysis from recipient mice, one month after transplantation, demonstrated hematopoietic engraftment of 88% or greater (Fig. 3b). Pulp, PDL and AvB cells were then extracted from recipient mice, cultured for 2 weeks and then either fixed and imaged or analyzed by flow cytometry. Presence of GFP^+^ cells can be detected in cultures from pulp, PDL and AvB (Fig. 3c) which demonstrates cells derived from transplanted CD45 enriched bone marrow. GFP^−^ resident cells can also be observed in the culture. Flow cytometric analysis demonstrates that 35–70% of pulp cells, 10–40% of PDL cells and 23–85% of AvB cells (n = 5–6) were GFP^+^. Representative images are seen in Fig. 3d. While this data confirms participation of CD45 enriched bone marrow cells in dental tissues, the contribution of HSCs specifically remains unaddressed.

### HSCs give rise to cells in pulp, PDL and AvB

**Figure 4:**
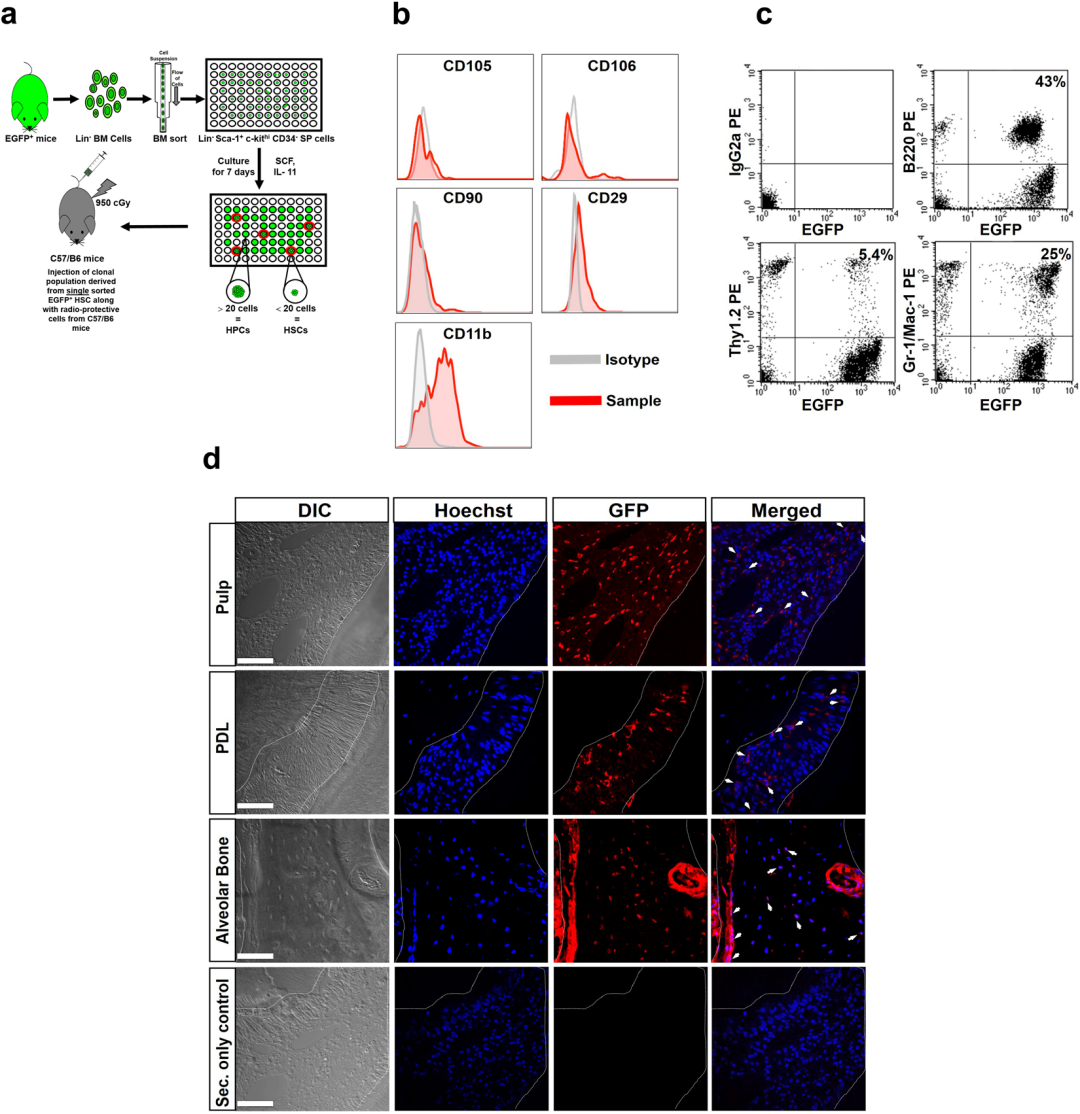
HSCs give rise to cells in pulp, PDL and AvB*:* (**a**) Schematic form of the transplantation method to generate mice with high-level, multilineage hematopoietic engraftment by a clonal population derived from a single HSC. (**b**) Representative flow cytometric analysis of Lin^−^Sca-1^+^C-kit^hi^CD34^−^SP cells for the presence of MSC markers. Images show that this population was negative for MSC markers such as CD105, CD106, CD90, CD29 (sample in red versus isotype in grey). These cells were positive for CD11b (Mac-1), confirming that the clonal population transplanted consisted of HSCs alone. (**c**) Representative flow cytometric analysis of the peripheral blood from a clonally engrafted lethally irradiated GFP^−^ recipient mouse shows GFP^+^ cells representing 43% of B cells, 5.4% of T cells and 25% of granulocytes-macrophages, 8 months after transplant. This indicates multilineage engraftment of the transplanted HSCs. (**d**) Representative images from section of the molar tooth from a transplanted mouse, examined after staining with the antibody to GFP (seen in red; Cy3). DIC images show cell morphology while nuclei are indicated by Hoechst stain in blue. Staining with GFP antibody demonstrates the presence of HSC-derived cells within pulp, PDL and AvB. This is more apparent in the merged images (shown by arrows). Most of the GFP^+^ cells are observed in the cell-rich layer of the pulp with a few GFP^+^ cells in the odontoblast layer surrounding the pulp. GFP^+^ cells were widely distributed along the length of the PDL. In the AvB, GFP^+^ cells can be visualized both in the osteoblasts lining the bone surface and in the osteocytes present in the bone matrix. Last panel shows the secondary only staining as control. Bar = 50 µM.

To unequivocally confirm that HSCs can differentiate into cells in pulp, PDL and AvB, we generated mice with high-level, multilineage hematopoietic engraftment by a clonal population derived from a single HSC as has been described previously^17,19,32,33^. The description of the method, given in Materials and Methods, is shown in schematic form in Fig. 4a. To confirm that the transplanted cells did not include any MSCs, we analyzed Lin^−^Sca-1^+^C-kit^hi^CD34^−^ population of cells for presence of MSC markers by flow cytometry. Figure 4b demonstrates that this population was negative for MSC markers such as CD105, CD106, CD90, CD29, thus confirming no MSCs were transplanted. These cells were positive for CD11b (Mac-1). It has been previously demonstrated that CD11b is expressed on activated HSCs^34^. This further confirms that the clonal population transplanted consisted of HSCs alone. Transplanted mice (n = 3) were analyzed for multilineage engraftment in the blood before being used for the experiment, as one of the hallmarks of a true HSC is its ability to reconstitute all lineages of blood. Figure 4c shows flow cytometric analysis of the peripheral blood from a representative recipient mouse exhibiting high-level multilineage engraftment 8 months after transplant. The percentage of total GFP^+^ cells within the different blood lineages in this mouse was 73%. A large number of granulocytes/macrophages (25%), and B cells (43%) in this mouse were GFP^+^. The slow turnover of T cells is believed to be responsible for the minority population of GFP^+^ T cells (5.4%). To investigate whether donor-derived cells are present in dental tissues, paraffin sections through molar tooth from clonally engrafted mice were evaluated by immunofluorescent staining. Analysis of images of sections stained with GFP antibody (visualized in red) demonstrates that a large number of cells within the pulp, PDL and AvB were GFP^+^ (marked by arrows), confirming the ability of HSCs to contribute to these dental tissues (Fig. 4d). Most of the GFP^+^ cells can be observed in the cell-rich layer of pulp with a few GFP^+^ cells in the odontoblast layer surrounding the pulp (Fig. 4d; pulp). GFP^+^ cells were widely distributed along the length of PDL (Fig. 4d; PDL). In the AvB, GFP^+^ cells can be visualized both in the osteoblasts lining bone surface and in the osteocytes present in bone matrix (Fig. 4d; AvB). These data confirm that HSC-derived cells can be found in the dental tissue region after transplantation.

### HSC-derived cells in pulp, PDL and AvB express dental tissue markers

**Figure 5:**
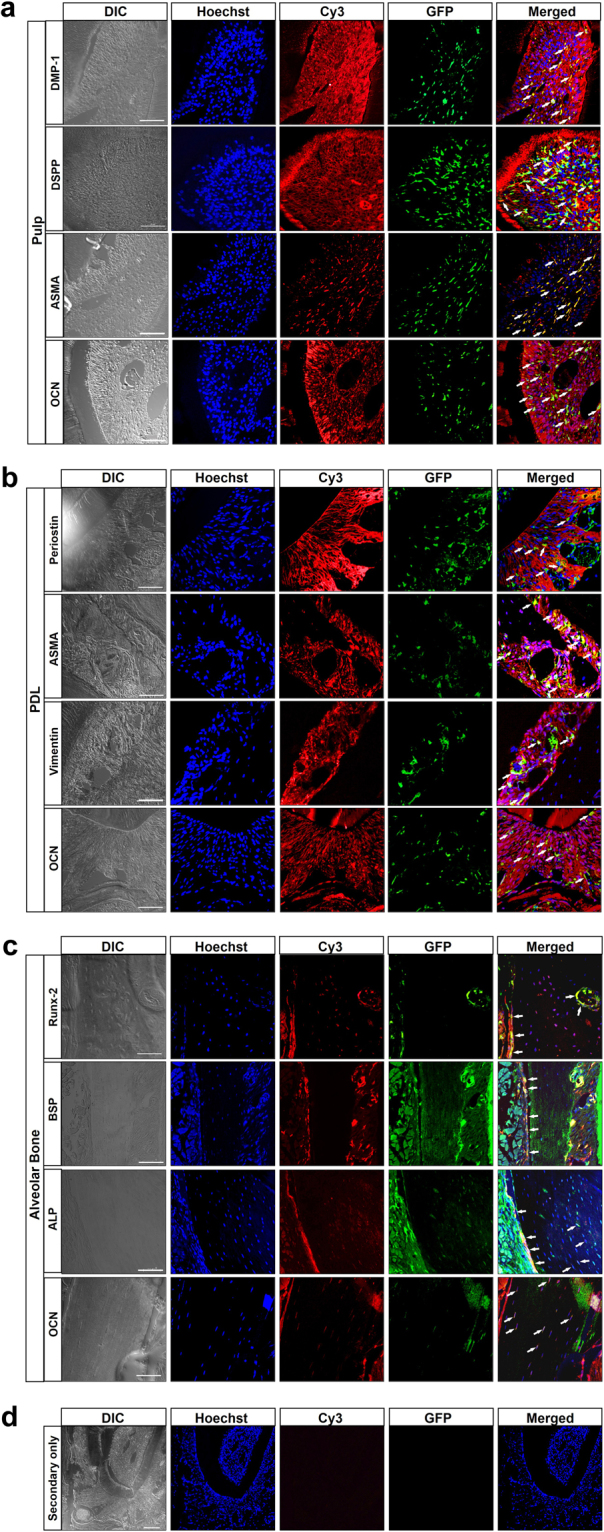
HSC-derived cells in pulp, PDL and AvB express dental tissue markers: Sections through mandible region, from mice transplanted with a clonal population derived from a single HSC, were stained with markers of the major resident cell populations present there and co-related with the GFP expression. (**a**) DMP-1, DSPP, ASMA and osteocalcin (OCN) were utilized for pulp; (**b**) periostin, ASMA, vimentin and OCN were utilized for PDL; (**c**) Runx-2, BSP, ALP and OCN were utilized for AvB. Secondary only staining is shown in (**d**). In all, DIC images show cell morphology, nuclei are indicated by Hoechst stain in blue, GFP staining is in green (Alexa Fluor 488) and the various markers are shown in red (Cy3). Co-staining of GFP and markers is seen in yellow indicated by arrows. In the pulp, cells co-expressing GFP and either DMP-1, DSPP and OCN can be seen within the pulp and only a very few in the odontoblast layer while cells co-expressing GFP and ASMA are seen only within the pulp layer. In the PDL, co-expression of GFP and periostin (seen here mainly in the extracellular matrix), ASMA, Vimentin and OCN can be seen throughout the entire length of the PDL. In AvB, co-localization of GFP with Runx-2 and BSP is mainly detected in the osteoblasts lining the surface of the bone while co-localization of GFP and ALP can be seen in the lining osteoblasts as well as the osteocytes in the bone matrix. Co-localization of GFP and OCN is mainly observed in the osteocytes in the bone matrix. Bar = 50 µm.

To confirm that HSCs can differentiate into the resident cell populations present in these dental tissues, we stained the sections, obtained from mice transplanted with a clonal population derived from a single HSC, with markers of the major cellular populations present there and co-related their expressions with GFP expression. As mentioned above, the main cell populations in pulp are fibroblasts and odontoblasts while PDL consists of fibroblasts, bone and cementum cells and osteoblasts/osteocytes form the main cellular population in AvB. Thus, markers such as DMP-1, DSPP, ASMA, vimentin, periostin, Runx-2, BSP, ALP and osteocalcin were used to identify the cell populations as has been described in studies previously^24–30^ and also used for the experiments earlier. Figure 5 shows staining of sections through the molar crown from a mouse transplanted with clonal population derived from single GFP^+^ HSC with GFP (in green) and DMP-1, DSPP, ASMA and osteocalcin (in red) for pulp (Fig. 5a); periostin, ASMA, vimentin and osteocalcin (in red) for PDL (Fig. 5b) and Runx-2, BSP, ALP and osteocalcin (in red) for AvB (Fig. 5c). In pulp, cells co-expressing GFP and either DMP-1, DSPP and OCN can be seen mainly within the cellular layer of the pulp and only a very few in the odontoblast layer while cells co-expressing GFP and ASMA are seen only within the pulp layer. In the PDL, co-expression of GFP and periostin (seen here mainly in the extracellular matrix), ASMA, Vimentin and OCN can be seen throughout the entire length of the PDL. In AvB, co-localization of GFP with Runx-2 and BSP is mainly detected in the osteoblasts lining the surface of the bone while co-localization of GFP and ALP can be seen in the lining osteoblasts as well as the osteocytes in the bone matrix. Co-localization of GFP and osteocalcin is mainly observed in osteocytes in bone matrix. Control secondary only staining is shown in Fig. 5d. This demonstrates that the donor-derived bone marrow HSCs can directly differentiate into resident cell populations found in pulp, PDL and AvB.

### Hematopoietic-derived cells in pulp, PDL and AvB can deposit collagen and undergo osteogenic differentiation

**Figure 6:**
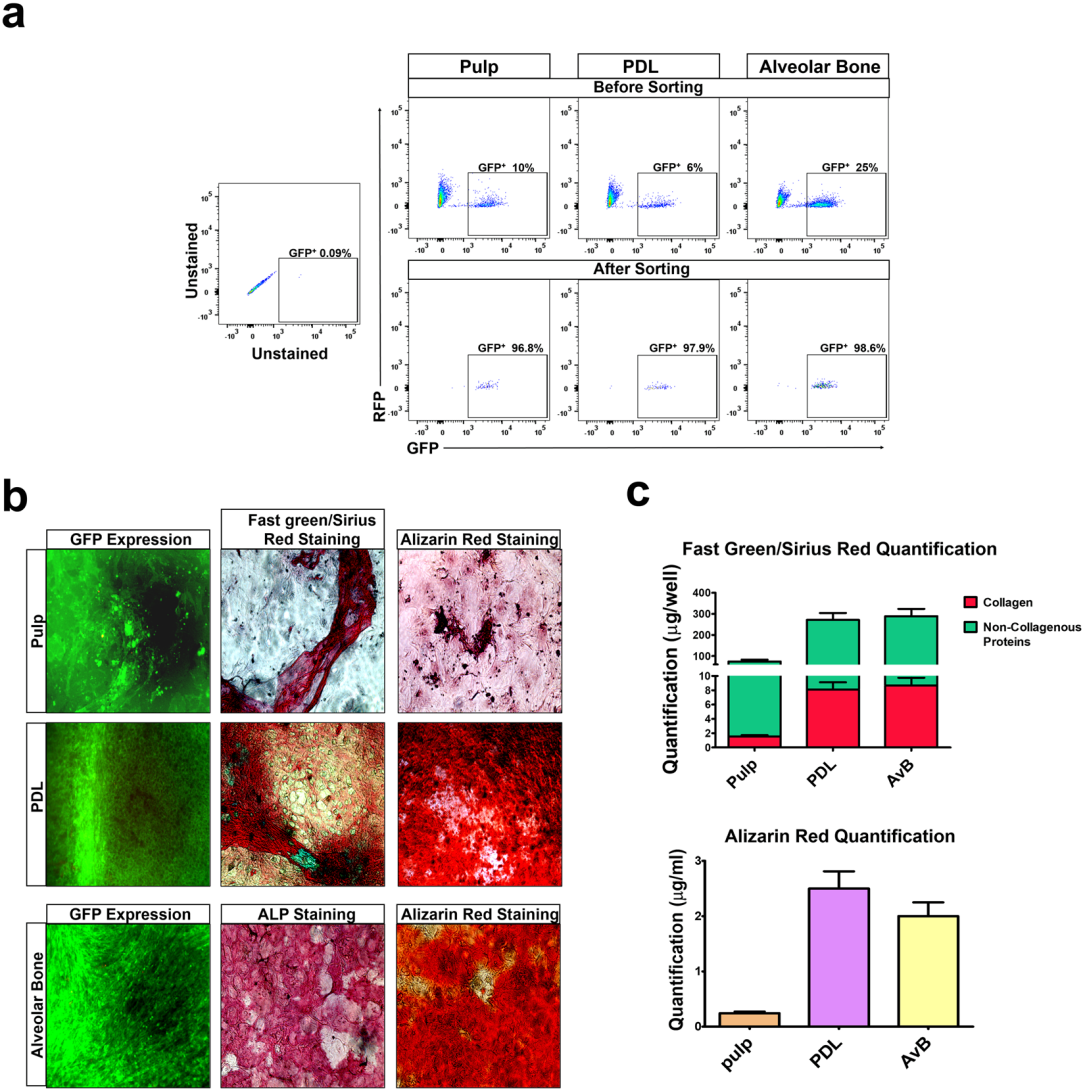
Hematopoietic-derived cells in pulp, PDL and AvB can deposit collagen and undergo osteogenic differentiation: Cells were obtained from the pulp, PDL and AvB of VavR mice by enzymatic digestion and grown till confluence. (**a**) They were sorted based on the GFP expression (gating was done using cells from a C57BL/6 mouse as negative controls-unstained). Upper panels show the percentage of GFP^+^ cells in the pulp (10%), PDL (6%) and AvB (25%). Lower panel showing the post-sort analysis of the cells demonstrates that a highly pure population of GFP^+^ cells was obtained (97–99%). (**b**) The GFP^+^ cells were then grown in regular and osteogenic media (described in Material and Methods) for 3 weeks, imaged and then stained with Fast Green/Sirius Red (cells in regular media) and Alizarin red (cells in osteogenic media). Merged images of GFP and RFP expression are shown in the left panel. Only GFP expression can be visualized indicating that no RFP expressing cells were present in the culture at the time of staining. Pulp cells deposit collagen as evidenced by pink staining (middle panel) and differentiate in culture, secreting a mineralized matrix as evidenced by calcium deposition indicated by the red staining (right panel). PDL cells demonstrate the formation of a honeycomb like structure which deposited both collagen (pink) and non-collagenous proteins (middle panel) and undergo mineralization and deposit calcium as evidenced by the red staining (right panel). AvB cells also showed the formation of bone like honeycomb structure and stained positive for ALP indicating the presence of osteoblasts (middle panel) which mineralized in osteogenic media and deposited calcium (right panel). (**c**) Quantification of the collagen and non-collagenous proteins and the calcium deposition, confirms the visual findings and strengthens the fact that hematopoietic-derived cells are functional.

In order to verify that the hematopoietic-derived cells in the dental tissues were functional, we performed *in vitro* analysis for collagen deposition and examined the osteogenic differentiation. As we have shown above, the major cell populations in pulp, PDL and AvB which have a hematopoietic origin are the fibroblasts and the osteoblasts. A major functional hallmark of fibroblasts is their ability to deposit collagen^35^ while osteoblasts in culture can differentiate and mineralize and deposit calcium^36^. We measured the collagen and non-collagenous proteins using the Fast Green/Sirius Red kit while deposition of calcium was measured by Alizarin red staining. For these experiments, we obtained the cells from the pulp, PDL and AvB of VavR mice as described in Material and Methods. On confluence, the cells were collected and sorted based on their GFP expression, as shown in Fig. 6a. The upper panels indicated the percentage of GFP^+^ cells present in each of the dental tissues, which was then sorted based on the gates shown. The lower panels indicates the post-sort analysis to ascertain the purity of the sorted cells. The percentage ranged from 97% to 99%, indicating that a highly pure population of GFP^+^ cells was obtained. The GFP^+^ cells were then grown in regular and osteogenic media for 3 weeks, after which Fast green/Sirius Red and Alizarin Red staining was done, as described in the material and Methods. The left panel in Fig. 6b shows the merged images displaying the GFP and RFP expression in the cells. As seen in these images, only GFP expression can be visualized indicating that no RFP expressing cells were present in the culture at the time of staining. Even though the number of cells was less in the pulp cultures, they do deposit collagen as evidenced by the pink staining (Fig. 6b, pulp, middle panel). These hematopoietic-derived cells can be made to differentiate in culture and secrete a mineralized matrix as evidenced by calcium deposition indicated by the red staining (Fig. 6b, pulp, right panel). Images from the PDL demonstrate the formation of a honeycomb like structure (resembling bone) which deposited both collagen (pink) and non-collagenous proteins (Fig. 6b, PDL, middle panel). In osteogenic media, the hematopoietic-derived PDL cells undergo mineralization and deposit calcium as evidenced by the red staining (Fig. 6b, PDL, right panel). AvB also showed the formation of bone like honeycomb structure and stained positive for alkaline phosphatase indicating the presence of osteoblasts (Fig. 6b, AvB, middle panel), which deposited collagen and non-collagenous proteins (image not shown). These hematopoietic-derived AvB cells mineralized in osteogenic media and deposited calcium (Fig. 6b, AvB, right panel). The quantification of the visual data is shown in Fig. 6c. As visualized, the amount of collagen and non-collagenous proteins as well as calcium deposited by pulp cells is less than the PDL and the AvB, but all three dental tissues do show quantifiable amounts indicating that these hematopoietic-derived cells are functional.

## Discussion

Advances in tissue engineering offers exciting translational opportunities for new therapies for dental regeneration. There has been considerable interest in recent years in the use of stem cells for repair of dental tissues such as pulp, PDL and AvB. Increasing evidence suggests that stem cells from sources such as dental tissue itself (PDL stem cells, pulp stem cells), stem cells from human exfoliated deciduous teeth, stem cells from apical papilla and dental follicle precursor cells^37,38^, adipose tissue^39^ as well as bone marrow can differentiate into cells in pulp and periodontium^1–3,5,7,8^. Zhou *et al*.^13^ reported that bone marrow derived cells preferentially migrate into pulp and periodontium over other organs and a small fraction of these migrating cells became dental tissue specific stem cells with a greater proliferative potential than resident stem cells. Thus, bone marrow can be a beneficial source of stem cells for dental repair. Bone marrow contains two types of stem cells, MSCs and HSCs. It is mostly believed that MSCs are responsible for the generation of tissue stromal cells but studies from our group have shown that HSCs can give rise to many types of mesenchymal cells such as fibroblasts/myofibroblasts and adipocytes (reviewed in Ogawa *et al*.^15,16^). We have also demonstrated that HSCs can differentiate into osteoblasts and contribute to repair of non-stabilized fracture^19^.

In this study we demonstrate, for the first time to our knowledge, that cells in the pulp, PDL and AvB can have a hematopoietic origin and HSCs can differentiate into the resident cells present in these tissues. Cells positive for CD45 can be visualized in pulp, PDL and AvB. The low percentage of CD45^+^ cells seen in culture can be explained by the fact that cells are thought to lose the expression of CD45 when grown in culture^40^. To eliminate the problem of transient expression of hematopoietic markers and also to confirm the findings, we utilized the VavR transgenic mice in which all hematopoietic-derived cells are GFP^+^ while all other cells are RFP^+^^22^. Presence of GFP^+^ cells can be seen in the pulp, PDL and AvB which also stained positive for markers of the resident cells in these tissues such as DMP-1, DSPP, ASMA and osteocalcin for pulp, periostin, ASMA, vimentin and osteocalcin for PDL and Runx-2, BSP, ALP and osteocalcin for AvB as well as for CD45 (hematopoietic marker) in all the three regions. These data confirm that cells with hematopoietic origins do exist in the pulp, PDL and AvB. To validate whether transplanted HSCs can differentiate into cells in dental tissues, we initiated the studies by transplanting lethally irradiated mice with CD45^+^ bone marrow cells and then to unequivocally confirm this, with a clonal population of cells derived from a single HSC. Both these data demonstrate that transplanted HSCs can differentiate into resident cells of dental tissues as confirmed by presence of cells which expressed GFP and markers such as DMP-1, DSPP, ASMA, vimentin, periostin, osteocalcin, BSP, ALP and Runx-2 in the pulp, PDL and AvB. We also demonstrate the functionality of these cells by showing that they have the ability to lay down collagen *in vitro* and undergo osteogenic differentiation depositing calcium. Collectively, all our data confirms that functional cells having a hematopoietic origin are present in dental tissues. A few previous reports demonstrating presence of GFP^+^ CD45^+^ cells in close association with reparative dentin^21^, c-kit enriched bone marrow cells giving rise to odontoblast like cells^41^ and CD34^+^ cells being discovered in human gingiva^20^ are in agreement with our observation that HSCs can contribute to the dental architecture, nonetheless, ours is the first study to describe this thoroughly. Most notable of the previous studies is the one by Frozoni *et al*.^21^ in which the authors show the presence of GFP^+^ cells around the reparative dentin in a parabiosis model. They speculate that it is “possible that the GFP^+^ cells around the reparative dentin represent new odontoblast-like cells derived from either a small population of mesenchymal stem cells (CD45^−^ of approximately 4.3%) or hematopoietic cells (CD45^+^ of approximately 54%) in the circulating peripheral blood”. However, they further comment that, “due to the lack of contribution of the hematopoietic cells to the regeneration of the cells in skeletal tissues as shown previously, the GFP^+^ cells around the reparative dentin most likely do not represent odontoblasts derived from hematopoietic cells”. They also do not do any additional characterization of these GFP^+^ cells to examine their origin. In addition, they have used a parabiosis model. This experimental approach cannot distinguish between the hematopoietic cells and the stromal cells which might be the origin of the GFP^+^ cells seen around the reparative dentin. In contrast, we have unequivocally demonstrated the presence of hematopoietic-derived cells in the dental tissue by using techniques which can clearly distinguish between the hematopoietic-derived and stromal-derived cells such as CD45 staining, transgenic mice as well as transplantation of a clonal population derived from a single GFP^+^ HSC. We do observe a difference in the percentage of the GFP^+^ (hematopoietic-derived) cells seen by the different methods. Studies suggest the cells lose their CD45 expression in culture^40^ which can explain the lower percentage of CD45^+^ cells observed. For the transgenic VavR mice, we did observe a varying percentage of hematopoietic-derived cells in the various mice that we analyzed. We can speculate that the hematopoietic-derived cells are most probably involved in the replenishment and repair of the surrounding structures such as the dentin and periodontium occurring regularly. Thus, the varied percentage of hematopoietic-derived cells in each mouse probably depends on the requirement for these cells. In the mice transplanted with HSCs, the percentage of donor-derived cells in pulp, PDL and AvB depends on the percentage of engraftment of the HSCs in the recipient, in other words, to what percentage the HSCs have reconstituted the bone marrow hematopoietic cells. Also the recipient mice have been irradiated, which might contribute to the varying percentage observed.

The hematopoietic-derived cells in our study were predominantly seen in cellular layers of pulp and PDL. Thus, it seems possible that these hematopoietic-derived cells would need the stimulus of an injury to differentiate into dentin-secreting odontoblasts in pulp or osteoid-secreting cells in PDL. Along those lines, we demonstrate that these hematopoietic-derived cells can be made to differentiate and deposit calcium in culture. In addition, our data also demonstrates that these hematopoietic-derived cells are highly proliferative and express the stemness markers such as Nanog and Sox-2 (data not shown). Thus, we can speculate that these cells might be more pluripotent and might actively participate in the repair of the dental tissue (dentin or periodontium) in case of injuries or disease. In fact our previous study, examining the contribution of hematopoietic-derived cells to fracture healing utilizing the transplanted mice, demonstrates that the GFP^+^ cells are recruited to the site of the injury, in this case the fracture callus, where they can differentiate into osteoblasts which help in the fracture healing^19^. Thus, the next steps in this study would be to examine the function and contribution of these cells especially in cases of injury or disease such as pulp exposure or periodontitis. It would be interesting to investigate if, during injury and recovery, the hematopoietic-derived cells are recruited or activated to a greater extent than the surrounding stromal-derived ones as well as the percentage of contribution of the hematopoietic-derived cells relative to the stromal-derived ones. It would also be noteworthy to examine that if the percentage of HSCs is increased in the peripheral blood (using drugs such as AMD3100 for HSCs mobilization from the bone marrow^42^), can we possibly increase the percentage of hematopoietic-derived cells in the dental tissues which can then initiate enhanced healing of the dental tissues. Thus, further studies are needed to clearly identify the contribution of these hematopoietic-derived cells to the dental tissue homeostasis and turnover. Mechanisms that govern the recruitment and homing of these cells to the dental tissues also need to be elucidated.

Our goal, with this study, was to examine as well as confirm by various methods the presence of hematopoietic-derived cells in the dental tissues as well as the ability of the HSCs to give rise to the cells in the pulp, PDL and AvB. Another interesting extension of this study would be to examine when during the embryonic development do these hematopoietic-derived cells originate or contribute to the dental tissues. This will involve examining the tissues at various prenatal (embryonic) as well as postnatal stages, which would require a separate study to fully investigate the ontogeny of these hematopoietic-derived cells in the dental tissues.

Collectively, our data along with these other studies strongly support the potential of hematopoietic-derived cells in dental tissues and open an avenue where these cells can be therapeutically exploited for dental repair. Given that HSCs are a primitive cell population, modulation of hematopoietic-derived cells for dental tissue healing may provide a sustained source of precursors, leading to more robust healing in damaged pulp/dentin and PDL, which harbors the regenerative cells for repair of peridontium and AvB. Long-term, elucidation of methods for augmenting hematopoietic-derived cell participation will have to be conducted to realize their maximum potential in new HSC-based therapies for enhanced repair of dental tissues.

## Material and Methods

### Mice

Breeding pairs of transgenic EGFP mice (C57BL/6-CD45.2) were kindly provided by Dr. Okabe^43,44^ (Osaka University, Japan). Breeding pairs of congenic C57BL/6-CD45.1 mice as well as Vav-Cre (*B6.Cg-Tg(Vav1-cre)A2Kio/J)* and mTmG (*B6.129(Cg)-Gt(ROSA)26S or* ^*tm4(ACTB-tdTomato,-EGFP)Luo*^*/J)* strains for generation of VavR transgenic mice were purchased from Jackson Laboratories. Crossbreeding was set up with hemizygous Vav-cre and homozygous mTmG mice and peripheral blood was obtained from tails of the pups at 4 weeks, which was analyzed for presence of GFP^+^ cells by flow cytometry, indicating transgenic VavR pup. All mice were bred and maintained at Animal Research Facility of the Medical University of South Carolina and Veterans Affairs Medical Center. All aspects of animal research and experimental protocols have been performed in accordance with the guidelines and regulations set by the PHS Policy on Humane Care and Use of Laboratory Animals and the Institutional Animal Care and Use Committee of Medical University of South Carolina and Department of Veterans Affairs Medical Center (AR # 3338).

### Reagents

Antibodies for lineage negative (Lin^−^) selection (B220, Gr-1, TER-119, CD4 and CD8), for sorting (anti-Sca-1-PE, Anti-c-kit-APC, biotin-conjugated lineage panel antibodies (B220, Gr-1, CD3ε, TER-119) and streptavidin-conjugated APC-Cy-7) and for multilineage engraftment analysis (PE conjugated anti-B220, Thy-1.2, Mac-1 and Gr-1) along with the appropriate isotypes were purchased from BD Pharmingen. Sheep anti-rat IgG Dynabeads® were purchased from Invitrogen. Biotinylated murine anti-CD34 was purchased from eBiosciences. CD105, CD106, CD90, CD39, CD11b were from Biolegend. Murine GFP, CD45, ASMA, Vimentin, DMP-1, Runx-2, periostin, BSP and ALP antibodies were purchased from Abcam. Osteocalcin (OCN) antibody was from Takara Bio (Clontech). DSPP antibody was from Santa Cruz Biotechnology. All secondary antibodies were from Jackson ImmunoResearch Laboratories. CD45-APC for flow cytometry was purchased from eBiosciences.

### Extraction of cells from pulp, PDL and AvB

Molar teeth were extracted, scraped to obtain PDL tissue and then cut to obtain pulp tissue. For AvB, a section of mandible was cut into pieces before digestion. All the tissues were digested in 3 mg/ml Collagenase type I and 4 mg/ml Dispase (Worthington) in PBS for 1.5–2 h at 37 °C. Single cell suspensions were obtained and cultured in α-modification of eagles’ media (AMEM)/20% fetal bovine serum (FBS) (Gibco) with 1% penicillin/streptomycin (P/S; Gibco), 200 µM Ascorbic acid (AA; Wako) and 2mM L-glutamine (Sigma) for 7–14 days, before being analyzed by immunofluorescent staining or flow cytometry. For analysis of the functionality of the HSC-derived cells, the GFP^+^ cells from pulp, PDL and AvB were sorted. They were then plated in regular media (as described above) and in osteogenic media (regular media with β-glycerophosphate [10 mM; MP Biochemicals) and cultured for 3 weeks. The cells were then fixed and stained with either Fast Green/Sirius Red (cells grown in regular media) or Alizarin Red (for the cells grown in osteogenic media).

### Non-adherent bone marrow cell Transplantation

Bone marrow cells were flushed from tibiae and femurs of 10–14 week EGFP (C57Bl/6-CD45.2) mice, pooled and washed with PBS/0.1% bovine serum albumin (BSA). For NAC preparation, mononuclear cells (MNC) from bone marrow were separated by density gradient using Lympholyte-M (Cedarlane) and plated in a T75 flask in AMEM/20% FBS/1%P/S. Supernatant with NACs was collected on Day 4, washed with phosphate buffered saline (PBS) and cells counted. Recipient C57BL/6-CD45.1 mice were given a single 950-cGy dose of total-body irradiation using a 4 × 10^6^ V linear accelerator (lethally irradiated) and 1 million NACs were injected via tail vein. For engraftment analysis, peripheral blood was obtained from retro-orbital plexus of anesthetized mice one month after transplantation and donor-derived GFP^+^ cells analyzed. All mice used had engraftment levels of 80% and higher. Cells were extracted from engrafted mice as described above and analyzed by immunofluorescent staining and flow cytometry.

### HSC Transplantation

#### Cell Preparation for transplantation

Generation of mice exhibiting high level of multilineage engraftment from a single HSC was conducted as previously described^17,19,32,33^. Briefly, bone marrow cells were flushed from tibiae and femurs of 10–14 week EGFP mice (donors), pooled and washed with PBS/0.1%BSA. MNCs were isolated by gradient separation using Lympholyte-M then further enriched for Lin^−^ cells by negative selection using antibodies to B220, Gr-1, CD4, CD8, TER-119, and Dynabeads® sheep anti-rat IgG beads. Lin^−^ cells after staining with anti-Sca-1-PE, anti-c-kit-APC, biotinylated anti-CD34 and biotinylated lineage panel antibodies (B220, Gr-1, CD3ε, TER-119) followed by streptavidin-APC-Cy7, were resuspended at 1 × 10^6^/ml in Hanks balanced salt solution/2% FBS/10 mM HEPES/1% P/S with Hoechst 33342 (Sigma; 5 µg/ml) and incubated at 37 °C for 60 min. Cells were then washed, stained with propidium iodide (1 µg/mL), resuspended in PBS/0.1% BSA and sorted using MoFlo Cell Sorter (DakoCytomation). Appropriate isotype-matched controls were analyzed. Individual Lin^−^Sca-1^+^c-kit^hi^CD34^−^ side population cells were deposited into wells of 96-well culture plates (Corning). Eighteen hours after deposition, wells containing single cells were identified and marked. The cells were then incubated for 7 days at 5% CO_2_, 37 °C in media containing AMEM/20% FBS/1% deionized fraction V BSA/1 × 10^−4^ mol/L 2-mercaptoethanol/100 ng/mL stem cell factor/10 µg/mL murine interleukin-11 (R & D Systems). Because the majority of HSCs are dormant and begin cell division a few days after initiation of cell culture, clones consisting of less than 20 cells, after 7 days of culture, were selected. This method significantly enhanced efficiency of generating mice with high-level multilineage engraftment by a single HSC.

#### Transplantation and Engraftment Analysis

C57Bl/6-CD45.1 recipient mice were lethally irradiated as described above. For transplantation, contents from the wells (≤20 clonal cells) were injected via tail vein into mice along with 500 CD45.1 bone marrow Lin^−^ Sca-1^+^c-kit^+^CD34^+^ cells. These cells have been shown to be effective radioprotective cells during post radiation pancytopenia period^45^. After transplantation, mice were fed irradiated diet (Tekland Global Diets) and MilliQ water *ad libitum*. Neomycin (200 mg/300 ml) was added to the water in the first month after transplantation to prevent infection. For multilineage engraftment analysis, peripheral blood was obtained from retro-orbital plexus of anesthetized mice two months after transplantation and RBCs lysed (1X PharM Lyse; BD Pharmingen). Donor-derived GFP^+^ cells in T cell, B cell, granulocyte and monocyte/macrophage lineages were analyzed by staining with PE-conjugated anti-Thy-1.2, anti-CD45R/B220 and a combination of anti-Gr-1 and anti-Mac-1, respectively. Only those mice demonstrating high-level multilineage engraftment (>50%) were used. The mandible with teeth were dissected from mice and fixed in 4% paraformaldehyde solution overnight, and then kept in 70% ethanol.

### Decalcification, Embedding and Sectioning

Decalcification was carried out using ethylenediaminetetra-acetic acid (EDTA; Lonza). The fixed mandible with teeth was put in 0.12 M EDTA for 14–21 days with daily weighing of sample and changing of solution. Decalcification end point was determined as the time when weight gain was noted^46^. Samples were washed with water, dehydrated through serial ethanols (70%, 95%, 100%), infiltrated (using Histoclear in place of xylenes to preserve EGFP expression) in serial Histoclear:paraffin solutions (1:0, 3:1, 1:1, 1:3, 0:1) and embedded under vacuum (60 °C). Five micron sections were generated.

### Immunofluorescent staining

To ensure that EGFP expression detected was not due to auto-fluorescence, an antibody against GFP was used with antigen retrieval. For antigen retrieval, sections were deparaffinized, and treated with 10% formic acid (or 2N HCl for CD45) before staining. Sections were permeabilized in 0.02%Triton X-100/PBS, blocked in 3%BSA/5% normal donkey serum/PBS, and then incubated with appropriate primary antibodies in PBS. After incubation, sections were again washed and incubated with appropriate fluorochrome-conjugated secondary antibodies in PBS, followed by Hoechst staining. For cells, inherent GFP was visualized without any staining. No antigen retrieval was done before staining.

### Fast Green/Sirius Red Staining

For the Fast Green/Sirius Red staining (Chondrex), the cells were fixed with Kahle fixative (as mentioned in the protocol with the kit) for 10 min and then washed with PBS. The cells were then incubated with the dye solution for 30 minutes, washed well with water and imaged under the inverted microscope. After imaging, the dye was extracted using the dye extraction buffer and the eluted dye solution was read at 540 nm and 605 nm. The amount of collagen and non-collagenous proteins were calculated as described in the protocol with the kit.

### Alizarin red staining

For Alizarin Red staining (Millipore), the cells were fixed with 4% paraformaldehyde and washed with PBS. They were then incubated with the alizarin red solution for 10 min, washed well with water, dried and imaged with the inverted microscope. After imaging the dye was extracted using the cetylpyridinium chloride extraction method and OD at 570 nm was read. The amount of alizarin red staining was calculated using a standard curve.

### Microscopy

Imaging was conducted using inverted and upright 80i microscope and A1R+ A1+ confocal microscope (Nikon) and processing was performed using Adobe Photoshop CS5 (Adobe Systems).

### Flow Cytometry

Cells were stained with antibodies for either CD45-APC, ASMA (pulp and PDL) or osteocalcin (AvB) with counter staining with APC-conjugated secondary antibody for ASMA and osteocalcin. Inherent GFP was also examined. For staining for ASMA and osteocalcin, which are intracellular, the cells were permeabilized using the Cytofix/Cytoperm kit (BD biosciences). Cells were analyzed using BD Accuri or BD LSRFORTESSA flow cytometer (BD Biosciences).

All experimental protocols were approved and performed in accordance with the relevant guidelines and regulations of Medical University of South Carolina.

### Data availability

All data generated or analyzed during this study are included in this published article.
